# Mathematical Modeling and Optimization of Fused Filament Fabrication (FFF) Process Parameters for Shape Deviation Control of Polyamide 6 Using Taguchi Method

**DOI:** 10.3390/polym13213697

**Published:** 2021-10-27

**Authors:** Zohreh Shakeri, Khaled Benfriha, Mohammadali Shirinbayan, Mohammad Ahmadifar, Abbas Tcharkhtchi

**Affiliations:** 1 Arts et Metiers Institute of Technology, CNAM, PIMM, HESAM University, F-75013 Paris, France; khaled.benfriha@ensam.eu (K.B.); mohammad.ahmadifar@ensam.eu (M.A.); 2 Arts et Metiers Institute of Technology, CNAM, LCPI, HESAM University, F-75013 Paris, France; Mohammadali.shirinbayan@ensam.eu (M.S.); abbas.tcharkhtchi@ensam.eu (A.T.)

**Keywords:** Taguchi design, ANOVA, FFF, response surface, cylindricity, process optimization

## Abstract

Fused filament fabrication (FFF) is a layer-by-layer additive manufacturing (AM) process for producing parts. For industries to gain a competitive advantage, reducing product development cycle time is a basic goal. As a result, industries’ attention has turned away from traditional product development processes toward rapid prototyping techniques. Because different process parameters employed in this method significantly impact the quality of FFF manufactured parts, it is essential to optimize FFF process parameters to enhance component quality. The paper presents optimization of fused filament fabrication process parameters to improve the shape deviation such as cylindricity and circularity of 3D printed parts with the Taguchi optimization method. The effect of thickness, infill pattern, number of walls, and layer height was investigated as variable parameters for experiments on cylindricity and circularity. The MarkForged^®^ used Nylon White (PA6) to create the parts. ANOVA and the S/N ratio are also used to evaluate and optimize the influence of chosen factors. As a result, it was concluded that the hexagonal infill pattern, the thickness of 5 mm, wall layer of 2, and a layer height of 1.125 mm were known to be the optimal process parameters for circularity and cylindricity in experiments. Then a linear regression model was created to observe the relationship between the control variables with cylindricity and circularity. The results were confirmed by a confirmation test.

## 1. Introduction

In the additive manufacturing process (AM), one of the fast prototyping methods, the CAD model is designed first and then made in 3D. The AM process is a layer-by-layer production process. Other names for this process are layer manufacturing, additive pro- cases, free-form fabrication, and solid free-form fabrication [1]. Three-dimensional printed parts show different properties depending on other AM techniques [2]. Fused deposition modeling (FDM), selective laser melting (SLM), multi-jet modeling (MJM), laminated object manufacturing (LOM), and stereolithography (SLA) and selective laser sintering (SLS) are various additive manufacturing (AM) methods [3,4,5,6,7,8]. Additive manufacturing provides the ability to produce complex geometries that are difficult to produce by conventional methods without complex tooling. The usage of AM technology has risen in recent years. Today, the use of AM method has increased because it reduces post-processing, material wastes, lower costs, creates high customization manufacturing parts, and greatly reduces overall product development [9,10].

Fused filament fabrication (FFF) is the most common process, and it is a 3D printing process that has been extensively investigated to produce metal and thermoplastic structures [11]. Due to less waste of material, high quality, and low manufacturing cost it is a common extrusion-based process [12]. FFF is a material extrusion process, according to ISO/ASTM terminology [13]. Thermoplastics are the base material in the form of filament that is selectively deposited through a nozzle over a movable bed. Among the different thermoplastics, we can mention PLA, ABS, ASA, and Nylon, which are more common in AM [14]. One can note that the limitation of this method is dimensional and geometrical accuracy. The Stratasys Company introduced this technology, and the proprietary term fused deposition modeling (FDM) was established [13].

Polyamide is a semi-crystalline thermoplastic with repeated amide sequences in the polymer backbone and H bonds between neighboring polymer chains. It has good mechanical, electrical, and thermal properties. PA6, PA12, and PA66 are all varieties of this thermoplastic, depending on the monomers that make it up. They are employed in wind turbines and oil and gas. PA6 is a low-cost, widely used synthetic polymer with wide applications. In the automobile industry, these polymers are commonly used. To improve some of its characteristics, extensive research is continuously being done [15,16].

Various process parameters utilized in this technique have an impact on the quality of FFF produced components [17]. On the other hand, the geometrical tolerance of AM- 3D printed parts is mostly determined by the process parameters’ setting. The process parameter could achieve improvement of the quality of prototypes by making appropriate adjustments to manufacturing parameters [18,19,20].

Fisher [21] proposed the concept of design of experiments (DOE) in the 1920s. DOEs are a structured and systematic method of running and evaluating controlled experiments to identify the factors that influence output variables. Because each component is independent of the others, this is a multivariable testing technique that varies them all simultaneously. DOEs define the specific setting levels of a couple of variables at which each run of the experiment will be carried out. For experimental planning, the Taguchi design method is a crucial tool. It provides a methodical and effective approach to cost, quality, and performance optimization. Taguchi [22] is the developer of the Taguchi design. A greater number of parameters may be evaluated at once in the Taguchi design method, and the optimal configuration can be reached with fewer resources than in the classic DOE approach. In fact, the main advantages of adopting Taguchi’s approach to design experiments with a simplicity of the experimental plan and the capability of studying interactions between multiple process parameters. The Taguchi orthogonal array (OA) is a basic fractional factorial design. It is a fractional orthogonal design based on a design matrix that lets users evaluate a set of many factor combinations at several levels. The Taguchi L9 [23,24] orthogonal array is a good experimental design approach with a small number of tests.

The response surface method is also one of the DOEs that examines the effect of different parameters on responses. This method helps to improve responses through a set including regression analysis and parameter optimization [25]. Many researchers have studied the effect and optimization of FDM process parameters on the shape deviation in FFF. Also, simple specimens and experimental designs such as Taguchi, ANOVA, and others are used in experimental investigations [26].

For example, Lee and Abdulla [27], using the Taguchi method, investigated the optimal elastic performance of a piece of ABS produced by 3D printing to achieve the maximum throwing distance from the prototype. They concluded that FDM variables such as raster angle, air gap and layer thickness have a major effect on the compliant ABS prototype’s elastic behavior.

Alafaghani and Qattawi [28], utilized the Taguchi technique to study the effects of infill density, infill pattern, layer height, and extrusion temperature in terms of the mechanical properties and dimensional accuracy of the FDM process with PLA filament. The results indicate that a lower extrusion temperature, smaller layer thickness, lower infill density, and hexagonal infill pattern will improve the dimensional accuracy. Also, with optimal layer thickness, higher extrusion temperature, and a larger infill density and triangle infill pattern, the strength of FDM parts is at a maximum.

Anitha and Arunachalam [18], using the Taguchi method (L18 orthogonal array), examined parameters such as road width, speed deposition, layer height on surface roughness. They observed that layer height, followed by road width and deposition speed, had the greatest influence on surface roughness.

Rizea and Anghel [29] investigated the effect of three critical parameters, including layer thickness, infill density, and orientation, on flatness and dimensional accuracy of Z-ULTRAT parts produced under FDM using the Taguchi method and L9 orthogonal array. Finally, they found that the effect of layer thickness and infill pattern was more significant than the orientation on shape deviation.

Sood and Ohdar [30], used the central composite design method, which is one of the DOE methods, and ANOVA analysis to optimize the parameters to investigate the impact of process parameters on specimen mechanical strength. Five basic process settings were studied, including raster angle, orientation, raster width, layer thickness and air gap and their impact on specimen responses, including tensile, flexural, and impact strength. Small raster angle, thick raster, lower number of layers and zero air gap will increase the mechanical strength.

Sheth and George [31] comprehended that spindle speed, feed rate, and the interaction between them have significant effects on cylindricity. They concluded that at lower spindle speeds, cylindricity is minimum.

Das and Mhapsekar [32], have evaluated the effect of FDM process parameters on optimization for the cylindricity tolerances with build orientation which minimize the support contact area. The circularity error is reduced with lower infill density. Also when the circular object is oriented with the vertical axis as the center and the base with the horizontal axis, the circularity error is minimal.

Aslani and Chaidas [33], applied the Taguchi method (L9) to estimate the effect of wall thickness and extraction temperature on dimensional accuracy and the surface quality of PLA parts under the FFF process. According to the results obtained, they understood that the dimensional accuracy and surface quality of PLA is improved and optimized using high-temperature extraction and average wall thickness values.

Chang and Huang [34] have worked on the optimization of raster width, contour width, raster angle, and contour depth variables for the optimization of flatness and cylindricity in FDM parts. The contour depth has the greatest influence, according to the ANOVA analysis of individual process parameters. Contour width is the second most important parameter, and raster width and raster angle have the least values.

Prakash and Sivakumar [35], investigated the effect of three parameters: filling density, horizontal and vertical orientation on the circularity of ABS parts 3D printed by the FDM method. It should be noted that they used the Taguchi method. They found that when a circular object is oriented with the vertical axis as the center and the horizontal axis as the base, the error on circularity is reduced compared to other orientations. The circularity of the parts is more influenced by the horizontal orientation.

Doloi and Kumar [36], applied the Taguchi approach to determine the impact of several process parameters on circularity error and diametrical deviation of ABS parts produced by FDM, including layer thickness, bed temperature, extruder temperature, infill density, and speed. From the results, it was found that in low bed temperature and moderate layer thickness, diametrical variation decreased. Also, the lowest circularity error was obtained at high speed, lower layer height, lower extruder temperature, and moderate infill density.

Using the Taguchi approach, Nagendra and Vikas [37], investigate how infill pattern, layer height, build orientation, and infill density impacted dimensional accuracy (DA), flatness, and cylindricity. An analysis of variance was used to assess the influence of process factors.

Saqib and Urbanic [38] investigated the influence on component accuracy on the FDM process by process variables with geometric shapes. In fact, to investigate the most influential process variables on the deformation of printed 3D parts, they designed experiments on flatness and circularity. The layer height, work envelope, and orientation were all investigated. The variation in cylindricity was maximum at 90° orientations, according to the results.

One of the most critical problems in the manufacturing of polymer parts is deformation during the process, which depends on the material’s rheological behavior. Also, 3D printing as one of the manufacturing methods is deformed due to the presence of micro voids in the interfaces of the deposited layers and layered structure of the 3D printing. Therefore dimensional stability is an essential factor for the designer. One of the methods to control geometric accuracy and reduce the resulting errors is to optimize the process parameters. In this paper, the cylindricity and the circularity as geometric tolerances of PA6 parts fabricated by FFF with variable parameters such as infill pattern, wall layers, layer height, and thickness were analyzed. The S/N ratios and ANOVA were employed to analyze the significant impacts and find the optimal parameter for minimum cylindricity and circularity simultaneously. Regression model of cylindricity and circularity was developed to predict them and to examine the correlation between different variables, which determines the relationship between each response and process parameters.

## 2. Materials and Methods

**Figure 1:**
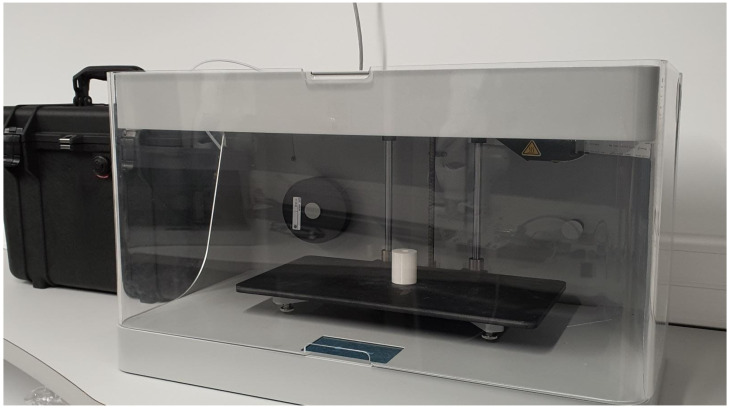
Markforged 3D printer.

**Figure 2:**
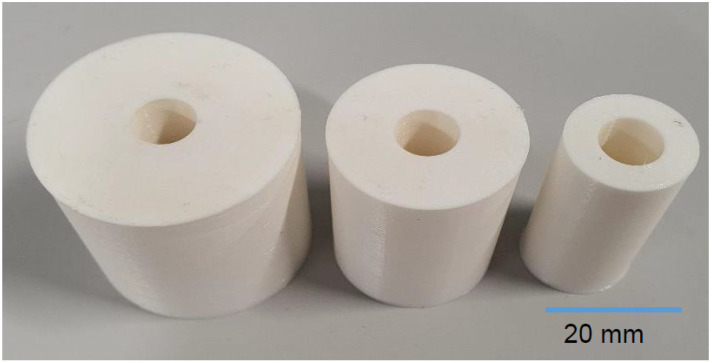
Hollow cylindrical parts.

In this research, the Markforged printer, one of the advanced FFF desktop 3D printers, has been used to 3D print the parts from polyamide 6 (Figure 1). Nylon White is a commercial material that Markforged Company (Watertown, MA, USA) developed. Nylon 6 or polycaprolactam are other names for polyamide 6 (PA6). It is one of the most widely used polyamides in the world due to its versatility. It also outperforms other polymers such as PLA and ABS in terms of mechanical properties, and the surface quality of PA 6 is excellent. Also, the 3D printer used is capable of printing limited materials such as PA 6, so this material was selected for experiments. As shown in Figure 2 for the experiments, hollow cylindrical parts with a fixed height of 40 mm and inner diameter of 10 mm, but variable outer diameter with the amounts of 20, 30, and 40 mm, were designed by CATIA-V5 software (V5, Dassault Systèmes, Paris, France), and STL files have been exported from it. The parts were printed using thermoplastic (polyamide 6) at a temperature of 273 °C. Table 1 represents the levels, and the process parameters that will be employed in the experiment. In this table the column of thickness shows the difference between the outer and inner diameter, and infill pattern identifies the structure and shape of the material inside of a part. Also, the thickness of each layer of deposited material is given by layer height, and wall layer indicates the thickness of the part’s walls. In this printer, some parameters have limitations, for example, for the layer height variable, the numbers 1, 1.125 and 2 can be selected. The selection of these parameters and the selection of different levels of each parameter primarily were performed based on the limitation of the variable parameters of the markforged 3D printer. Selected thicknesses were considered to observe both small and large dimensions of the responses. The number of wall layers was also selected based on the dimensions of the cylinder. Considering the volume required to apply different infill patterns, the number of one wall created an unsuitable surface in the cylinder. Table 2 shows the Taguchi orthogonal array that controls the parameter combinations for each experiment.

**Figure 3:**
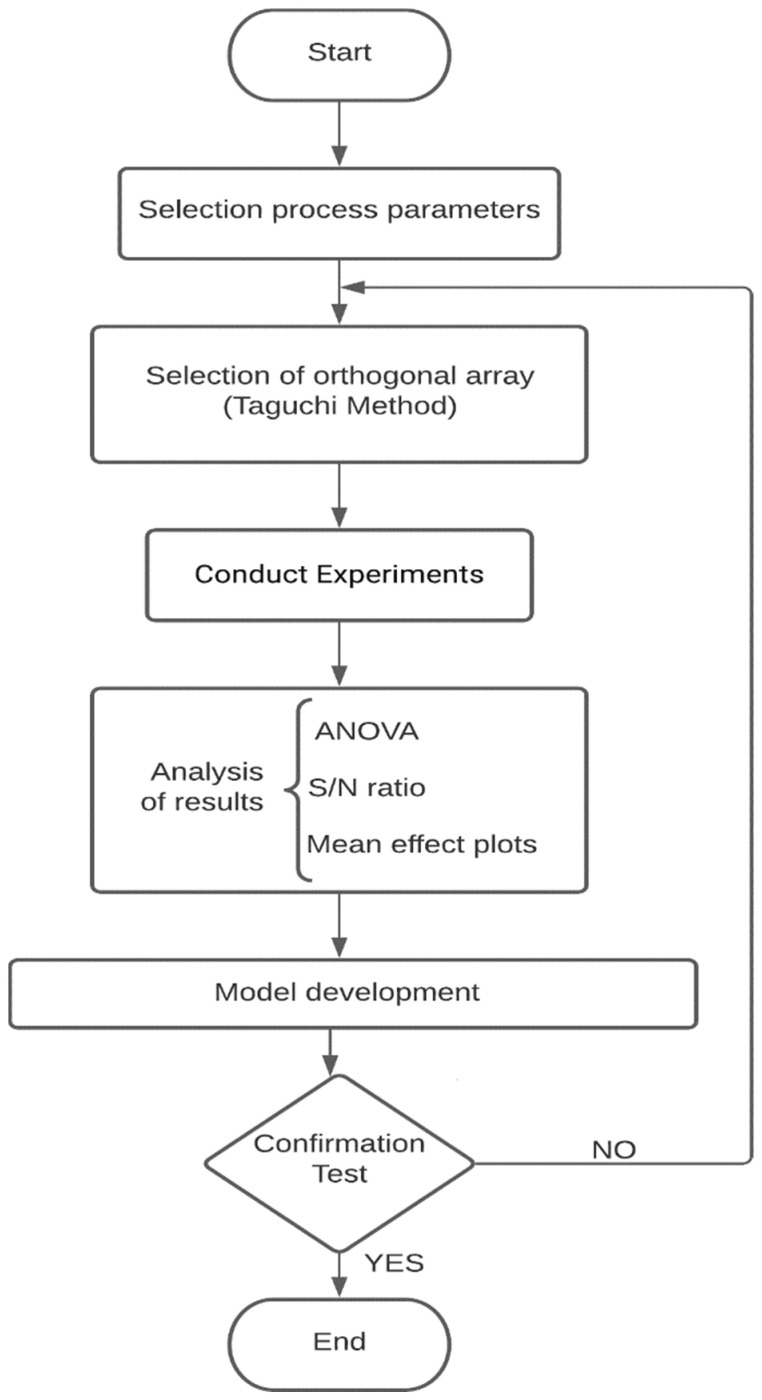
Flowchart of implementing the steps.

The standard modeling software CATIA-V5™ was used for 3D modeling. An STL file is extracted from the designed CAD model. Then, pieces were 3D printed, and all the parts were scanned with a 3D laser scanner named Solutionix D500 (Medit, Seoul, Korea). This professional scanner specializes in scanning small and detailed things and the most complex products. It has a resolution of 0.055 mm and an accuracy of 0.01 mm. Also, the scan speed in this scanner is high because of more powerful engines and enhanced algorithms. The 3D scanner scans the product from multiple angles automatically. A blue light reflects objects and reaches the camera lens, which obtains point-by-point coordinate measurements and geometry of the parts. The STL files were exported from the Solutionix ezScan which controls the Solutionix D500 scanner. The evaluation of shape deviation errors was undertaken with Geomagic^®^ Control X™ software (based on ASME Y14.5M standard, v2020.1.1, 3D Systems, Research Triangle, SC, USA) by comparing the CAD model whit STL files were exported from Solutionix ezScan™. On the other hand, The STL scanned file must be aligned with CAD using component alignment to obtain consistent results. At least four points of cloud data must be matched to the CAD model during the alignment process. The circularity and cylindricity were evaluated by using the standard ASME Y14.5M 19,941 The measured values of cylindricity and circularity are shown in Table 3. In Figure 3, each step in the flowchart will be used in the rest of the article.

## 3. Results and Discussion

### 3.1. Analysis of Experimental Data

S/N ratio and ANOVA were used to analyze data from the experiments. Experimental measured data of Table 3 for the cylindricity and circularity were analyzed by using the statistical software MINITAB 19.0^®^ (LLC, State College, PA, USA).

### 3.2. Analysis Using S/N Ratio

To analyze the effect of process variables on each response (cylindricity), the S/N ratio was utilized. When the experimental results were presented as S/N ratios, it was discovered that they varied linearly. For the data analysis, out of the different quality characteristics of S/N ratio, the ‘smaller is better’ was considered. S/N ratio (*η*) can be obtained by using Equation (1), where *MSD* stands for mean-square deviation, the average of the data points is indicated by the *Y*, and *Y*_0_ represents the target value, and *σ*^2^ is the variance. Equation (2) calculates the *MSD* value [30].

*η* = −10 *log*(*MSD*) (1)

*MSD* = *σ*^2^ − (*Y* − *Y*_0_)^2^ (2)

### 3.3. Response Table for S/N Ratio for Cylindricity and Circularity

These S/N ratios are added for each level of each parameter according to Taguchi’s procedures, and then their average is determined. The highest minus the lowest average is the delta statistic and the individual contribution of the parameters (Rank) is shown in Table 4 and Table 5.

### 3.4. Mean Affects Plots for S/N Ratios

**Figure 4:**
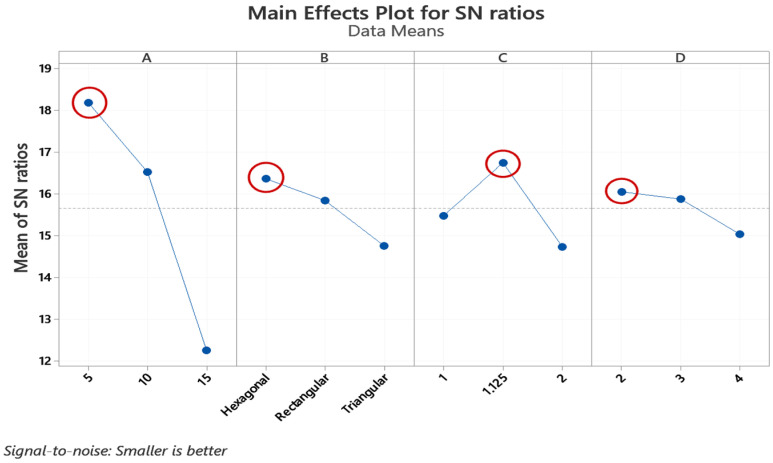
Main effect plot cylindricity. A, B, C, and D represent the Thickness, Infill Pattern, Layer height, and Wall Layer, respectively.

**Figure 5:**
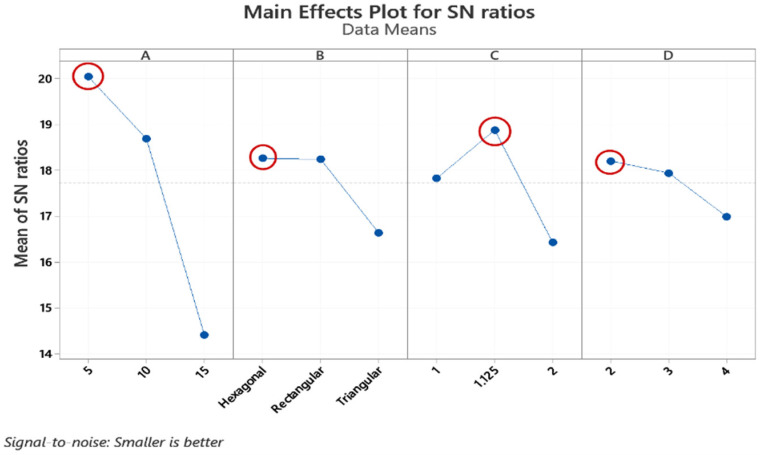
Main effect plot circularity. A, B, C, and D represent the Thickness, Infill Pattern, Layer height, and Wall Layer, respectively.

In Figure 4 and Figure 5, The S/N graphs were used to determine the optimal parameters in the form of average S/N ratios for cylindricity and circularity. As minimization of the output parameters is required, the smaller is better is selected to maximize mathematical expression for the S/N ratio for cylindricity and circularity. In Figure 4A–D and Figure 5A–D correspond to thickness, infill pattern, layer height, and wall layer, respectively. The horizontal axis shows the different levels for each parameter. According to the respective average S/N ratios, all phases of the given graphs show that in the 5 cm thickness, hexagonal infill pattern, 1.125 mm layer height, and two wall layers are the required 3D printing parameters for the best cylindricity and circularity values.

### 3.5. Analysis of Variance

The ANOVA method was used to determine the significance and contribution of each process parameter to the response variables. The results are shown in Table 6 and Table 7, where DF represents degrees of freedom, and Adj SS shows the adjusted sum of squares and can be calculated as shown in Equation (3) where *η_i_* shows the mean S/N ratio and *η_j_* shows the total mean of S/N ratio and *n* is total number of experiments. Adj MS is the adjusted mean sum of squares, F-Value and *p*-Value are the variance of the group means and probability value respectively. *p*-Value describes the significance level of each parameter, and it can be calculated as shown in the 4, where *SS_D_* is sum of squared deviations each process parameter and *SS_T_* is Total sum of squared deviations [30]. The findings reveal that the F-Value of thickness and infill pattern is greater than the F-Value of the wall layer and layer height. As a result, they have the greatest influence on cylindricity and circularity values.

(3) $$ss_{T}=\overset{n}{\underset{i=1}{\sum }}{\left(\eta_{i}-\eta_{j}\right)}^{2}$$

(4) $$P=\frac{s_{eq}ss_{D}}{s_{eq}ss_{T}}$$

### 3.6. Response Surface Regression Model

Since the Taguchi approach just analyzes the major factors which influence variables, without taking account of the correlation between them, response surface regression was used to determine the relationship between the control variables and response variables for polyamide 6. The purpose of the response surface method is to formulate the response as a function of contributing variables and to discover the best set of factor levels that provides the best response value depending on the research goals.

Constants and predictor coefficients made up the regression model. The linear response surface regression model is represented by Equation (5) [39].

(5) $$y=\beta_{0}+\beta_{1}x_{1}+\beta_{2}x_{2}+\ldots +\beta_{j}x_{j}+\varepsilon$$

where *x_i_* is the process parameter and *β* is the coefficient to be determined based on the experimental data (*β*_0_ = constant coefficients *, β*_1_, *β*_2_, …, *β_j_ =* linear coefficients) and ε describes the measurement error. The response y can be any of the output parameters. Models were developed by using the software MINITAB 19.0^®^.

Linear regression equation used in the estimation of cylindricity values:

cylindricity = −0.0260 + 0.01185 A + 0.0118 B + 0.0192 C + 0.0102 D(6)

Linear regression equation used in the estimation of circularity values:

circularity = −0.0317 + 0.00876 A+ 0.0090 B + 0.0365 C + 0.0088 D(7)

Here A, B, C and D are the factors that represent the thickness, infill pattern, layer height and wall layer, respectively. The above empirical model predicts the cylindricity and circularity for any combination of process parameters. The correlation coefficient or R-squared is a statistical measure that represents a dependent variable’s proportion of variation and usually is between 0 to 100%. In the preceding model of cylindricity and circularity, the values of R-squared is 86.40% and 84.67%, respectively, which demonstrates that the actual and predicted values have a good correlation.

**Figure 6:**
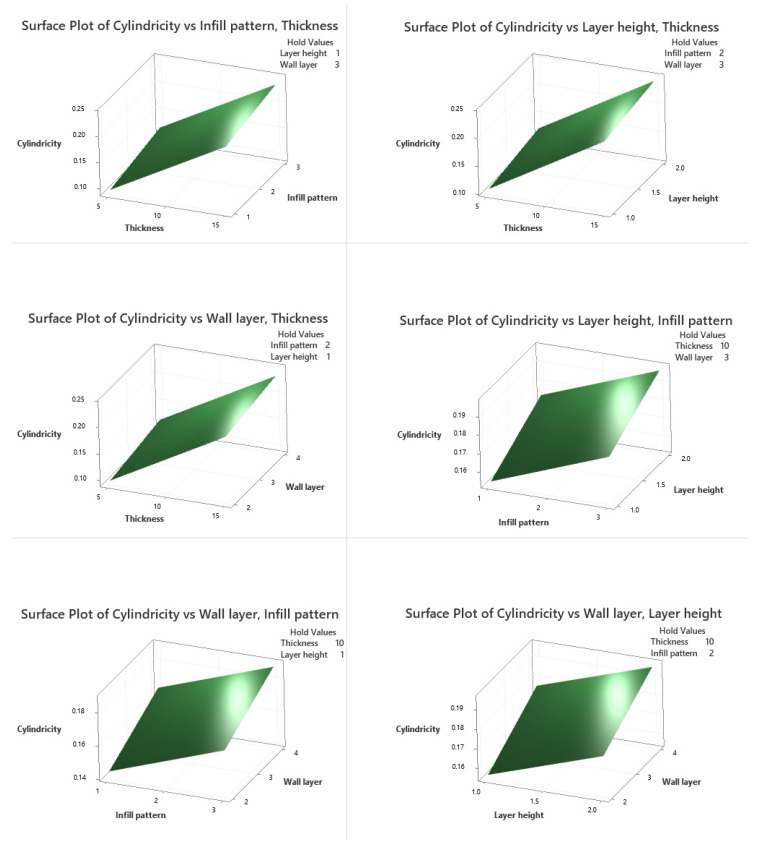
Cylindricity surface plots.

**Figure 7:**
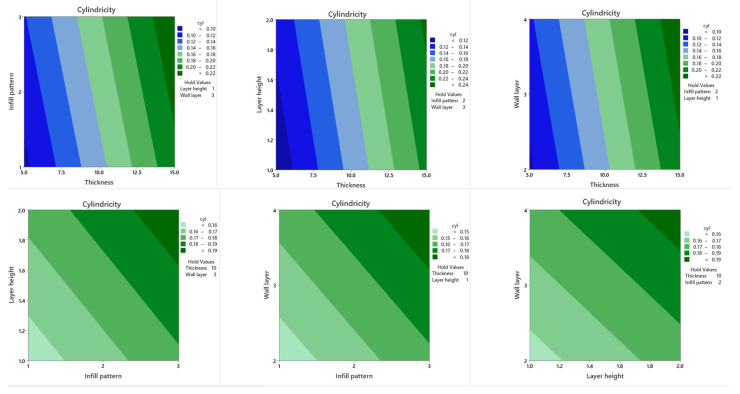
Cylindricity contour plots.

**Figure 8:**
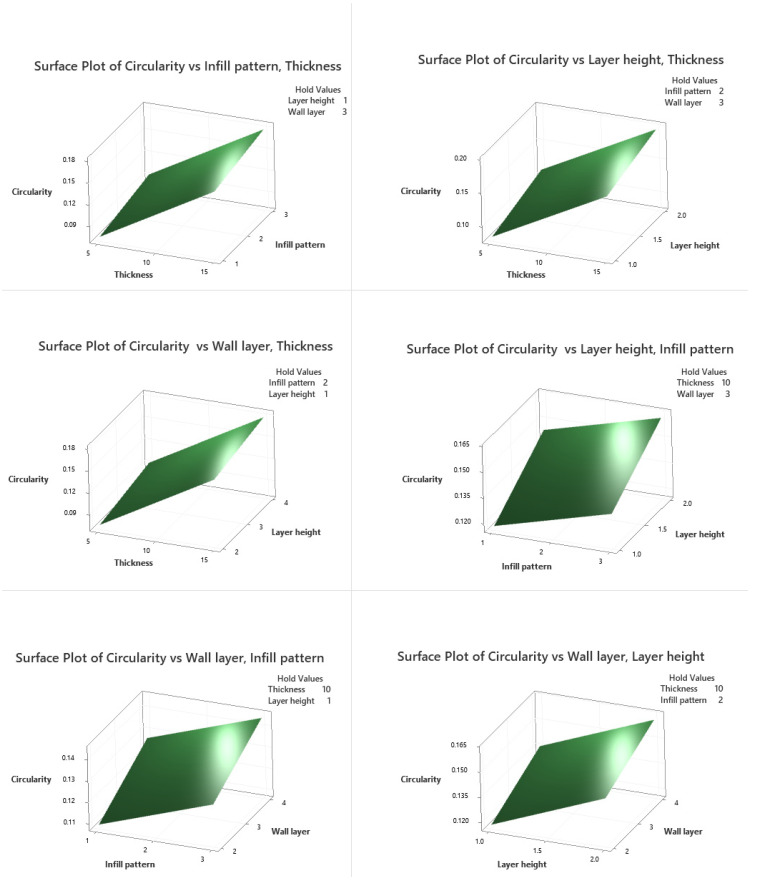
Circularity surface plots.

**Figure 9:**
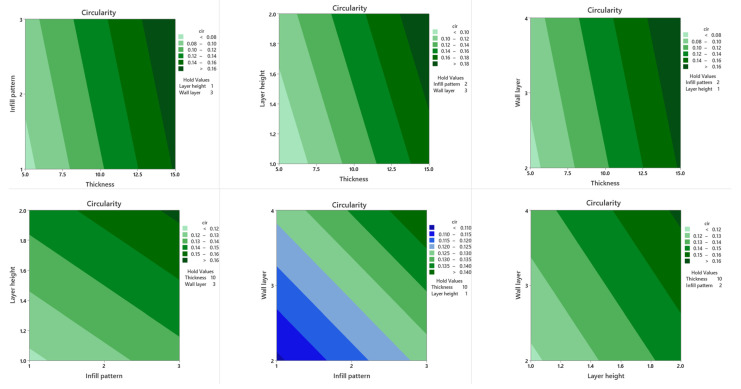
Circularity contour plots.

In a DoE study, response surface plots are extremely beneficial for evaluating the interaction effects between two parameters simultaneously on the responses. The contours of a response surface will be plotted to help visualize the shape of the response surface, and each contour corresponds to a certain response surface height. Response surface plots and contour plots of each two parameter combination on cylindricity (Figure 6 and Figure 7) and circularity (Figure 8 and Figure 9) are plotted when the other two parameters are held constant at their default values, as shown in the upper right area. As is shown in Figure 6 and Figure 7, cylindricity is plotted versus different levels. Contour plots show that cylindricity is minimal at low thickness values and hexagonal infill patterns (B). Similarly, from the interaction plot of the thickness and layer height on cylindricity at low values of thickness and layer height levels, cylindricity is minimum. Also, at low levels of thickness and levels of wall layer, cylindricity is low. Cylindricity is minimum at low layer height levels and hexagonal infill pattern. At low wall layer levels and hexagonal infill pattern, cylindricity is low. Also, cylindricity is minimal in low wall layers and low layer height values. The 3D surface plots show the same combination of process variables.

Contour and surface plots for circularity are shown against various levels in Figure 8 and Figure 9. From the interaction plot of the thickness and infill pattern, low values of the thickness, and hexagonal infill patterns, circularity is low. At lower thickness and layer height levels, circularity is also minimal. Circularity is low at low thickness levels and wall layers values. Circularity is minimal in lower layer height levels and hexagonal infill pattern. Also, lower wall layer levels with a hexagonal infill pattern provide lower circularity. Cylindricity is minimal in low wall layers levels and low layer height values. The 3D contour plots show the same combination of process variables.

Taguchi design was used to create reproducible and valid results to investigate the effect of selected parameters on the geometrical error. This study revealed many unexpected findings. The impact of each is further analyzed by the impact of parameters on the responses and compared with related sources:

1. Parts with higher thickness have more material mass than the smaller diameter parts, and this causes the gravity force, which is one of the forces affecting the deformation, to be higher, and it will cause more geometric error so that the lowest thickness is shown better responses.
2. As it turns out, the amount of geometric error is minimal in the hexagonal infill pattern. This could be due to the polymer nodes. The more nodes, the tighter the piece and the less deformation. Since the number of polymer nodes increases in the hexagonal pattern, the deformation is less than other infill patterns. In the research mentioned in the introduction, the effect of the infill pattern was studied, and similarly, the hexagonal infill was introduced as the optimal infill pattern [28].
3. As the layer height is decreased, the number of deposited layers increases, and this will cause more interfaces and adhesion will be reduced. On the other hand, a high layer height causes higher thermal gradients between the layers, and more deformation will be accrued, and geometrical errors will be increased. But as mentioned in the literature of the article with different materials, it was observed that as the layer height decreased, the shape errors decreased. The reason may be the type of material that is used in this article. However, the layer height study results in other materials such as ABS and PLA showed that the lower the layer height, the lower the geometric and dimensional accuracy at lower layer heights. This difference in results between other research and the current research can be due to the limitation of the 3D printer used because values less than 1 and more than 2 cannot be selected as layer height [28,36,40].
4. According to the results, it was found that the amount of cylindricity and circularity is less for a lower number of wall layers. This may be because, in 3D printing of parts, the wall layers are first deposited on the platform, and then the internal infill will be deposited. During this short period, the wall layer will be fully solidified. Therefore, we will not have good adhesion in the interface of walls and infill sections compared to the internal infill section. However, wall thickness (wall layer) studies on PLA revealed that the dimensional accuracy would be improved and optimized in average wall thickness values. The explanation for the variation in findings might be related to limitations in the design of the cylinders and 3D printers that were utilized. The type of material used may affect the results [33].

### 3.7. Confirmation Test

To validate the established empirical models, a confirmation experiment was carried out. The optimal process parameter condition was used to conduct a confirmation test. The optimal values for parameters were hexagonal infill pattern, two wall layers, 1.125 mm layer height, and 5 cm thickness. Therefore, a part with optimal parameters was 3 D printed, and the Solutionix D500 scanner measured the cylindricity and circularity values. Then the optimal values of the parameters were placed in the developed cylindricity and circularity formulas, and the obtained values were compared with the values obtained from the experiment. Table 8 shows these values. Because of the uncertainty, output response was predicted to fall within the confidence interval range. The confirmation test revealed that the suggested models for cylindricity and circularity were acceptable in 95% of the experimental domain’s confidence interval (CI). According to the results presented in Table 8, it was found that the error of cylindricity and circularity is less than 20%, which is acceptable [41]. The results of the confirmation tests indicate that the optimization was successful.

## 4. Conclusions

The present work leads to the following conclusions of experimental research on the effect of layer height, infill pattern, and the number of wall layers on shape deviation (cylindricity and circularity) in different thicknesses in the FFF process.

According to the L9 orthogonal array, the experiments were carried out by a MarkForged^®^ Mark Two 3D printer.

The cylindricity and circularity indicator was measured using a Solutionix D500 scanner and Geomagic^®^ Control X™ software. According to the results from ANOVA, the layer height and thickness influence is much more significant than the influence of the infill pattern and wall layers on cylindricity. Similarly, the effect of layer height and thickness on circularity is much more significant than the influence of the infill pattern and wall layers, according to DOE and optimization results.

Also, the results can help the designer to understand the phenomenological interactions between the parts’ dimensions and the evolution of the geometric tolerances. From the (S/N) analysis for the cylindricity and circularity, it was found that layer height of 1.125 mm, hexagonal infill pattern, 5 mm thickness, and two wall layers were the optimal process parameters to minimize shape deviations. Also, a regression model was developed, and a confirmation test was applied to show that the predictions are in good agreement with experimental data.

Three-dimensional printing results can be influenced by unstable machine conditions, operator error, and other factors. Regression models were established to predict cylindricity and circularity.

## Figures and Tables

**Table 1:** The process parameters and their levels.

Level	Thickness (mm)	Infill Pattern	Layer Height (mm)	Wall Layer
1	5	Hexagonal	1	2
2	10	Rectangular	1.125	3
3	15	Triangular	2	4

**Table 2:** L9 orthogonal array.

No. of Trial	Thickness (mm)	Infill Pattern	Layer Height (mm)	Wall Layer
1	5	Hexagonal	1	2
2	5	Rectangular	1.125	3
3	5	Triangular	2	4
4	10	Hexagonal	1.125	4
5	10	Rectangular	2	2
6	10	Triangular	1	3
7	15	Hexagonal	2	3
8	15	Rectangular	1	4
9	15	Triangular	1.125	2

**Table 3:** Measured values of cylindricity and circularity.

No. of Trial	cyl 1 (mm)	cyl 2 (mm)	Mean cyl (mm) (±St Dev)	cir 1 (mm)	cir 2 (mm)	Mean cir (mm) (±St Dev)
1	0.1112	0.1101	0.11065 (±0.0007778)	0.0864	0.0878	0.0871 (±0.0009899)
2	0.1047	0.1024	0.10355 (±0.0016263)	0.0853	0.0741	0.0797 (±0.0079196)
3	0.1648	0.1612	0.163 (±0.0025456)	0.1463	0.01376	0.14195 (±0.0937199)
4	0.1309	0.1294	0.13015 (±0.0010607)	0.1098	0.0975	0.10365 (±0.0086974)
5	0.1571	0.1528	0.15445 (±0.0030406)	0.1192	0.1204	0.1198 (±0.0008485)
6	0.1646	0.1644	0.1645 (±0.0001414)	0.1267	0.1262	0.12645 (±0.0003536)
7	0.2423	0.2444	0.24335 (±0.0014849)	0.2018	0.2015	0.20165 (±0.0002121)
8	0.26	0.263	0.2615 (±0.0021213)	0.1897	0.1943	0.192 (±0.0032527)
9	0.2287	0.2267	0.2277 (±0.0014142)	0.1798	0.176	0.1779 (±0.0026870)

**Table 4:** Response of variable parameters for cylindricity.

Level	Thickness (mm)	Infill Pattern	Layer Height (mm)	Wall Layer
1	18.19	16.37	15.48	16.06
2	16.53	15.85	16.75	15.88
3	12.26	14.76	14.74	15.04
Delta	5.93	1.61	2.01	1.02
Rank	1	4	2	3

**Table 5:** Response of variable parameters for circularity.

Level	Thickness (mm)	Infill Pattern	Layer Height (mm)	Wall Layer
1	20.04	18.27	17.83	18.21
2	18.69	18.25	18.89	17.95
3	14.41	16.64	16.43	16.99
Delta	5.63	1.63	2.45	1.22
Rank	1	3	2	4

**Table 6:** Response of variable parameters of cylindricity.

Source	DF	Adj SS	Adj MS	F-Value	*p*-Value
A	2	0.023508	0.011754	35.49	0.027
B	2	0.000841	0.000421	1.27	0.441
C	2	0.001806	0.000903	0.72	0.268
D	2	0.000662	0.000331	-	-
Pulled Error	2	0.000662	0.000331	-	0.559
Total	8	0.026818	0.01374	-	-

**Table 7:** Response of variable parameters of circularity.

Source	DF	Adj SS	Adj MS	F-Value	*p*-Value
A	2	0.013321	0.00666	28.51	0.034
B	2	0.000657	0.000328	1.41	0.416
C	2	0.0001749	0.000875	3.74	0.211
D	2	0.000467	0.000234	-	-
Pulled Error	2	0.000467	0.000234	-	0.584
Total	8	0.016194	0.008331	-	-

**Table 8:** Comparison of cylindricity and circularity predicted by ANOVA and confirmation experiment.

Shape Error	Predictions (±95% CI) (mm)	Experimental (mm)	Error (%)
cylindricity	0.08705 (±0.1733)	0.10261	15.1
circularity	0.06851 (±0.1367)	0.07822	12.4

## Data Availability

The data presented in this study are available on request from the corresponding author.
